# Retinal and brain damage during multiple sclerosis course: inflammatory activity is a key factor in the first 5 years

**DOI:** 10.1038/s41598-020-70255-z

**Published:** 2020-08-07

**Authors:** Irene Pulido-Valdeolivas, Magí Andorrà, David Gómez-Andrés, Kunio Nakamura, Salut Alba-Arbalat, Erika J. Lampert, Irati Zubizarreta, Sara Llufriu, Eloy Martinez-Heras, Elisabeth Solana, Nuria Sola-Valls, María Sepulveda, Ana Tercero-Uribe, Yolanda Blanco, Anna Camos-Carreras, Bernardo Sanchez-Dalmau, Pablo Villoslada, Albert Saiz, Elena H. Martinez-Lapiscina

**Affiliations:** 1 Service of Neurology, Department of Neurology, Center of Neuroimmunology, Hospital Clinic of Barcelona, Institut d’Investigacions Biomèdiques August Pi Sunyer (IDIBAPS), University of Barcelona, Villarroel 170, 08036 Barcelona, Spain; 2 Child Neurology Unit, Hospital Universitari Vall d’Hebron, Vall d’Hebron Research Institute (VHIR), EURO-NMD and RND-ERN, Passeig de la Vall d’Hebron 119-129, 08035 Barcelona, Spain; 3 Department of Biomedical Engineering, Lerner Research Institute, Cleveland Clinic, 9500 Euclid Avenue, Cleveland, OH 44195 USA; 4 Cleveland Clinic Lerner College of Medicine, 9500 Euclid Avenue, Cleveland, OH 44195 USA; 5 Service of Ophthalmology, Hospital Clinic of Barcelona, Institut d’Investigacions Biomèdiques August Pi Sunyer (IDIBAPS), University of Barcelona, Villarroel 170, 08036 Barcelona, Spain

**Keywords:** Neurology, Neurological disorders, Demyelinating diseases, Multiple sclerosis

## Abstract

Understanding of the role of focal inflammation, a treatable feature, on neuro-axonal injury, is paramount to optimize neuroprotective strategy in MS. To quantify the impact of focal inflammatory activity on the rate of neuro-axonal injury over the MS course. We quantified the annualized rates of change in peripapillary retinal nerve fiber layer, ganglion cell plus inner plexiform layer (GCIPL), whole-brain, gray matter and thalamic volumes in patients with and without focal inflammatory activity in 161 patients followed over 5 years. We used mixed models including focal inflammatory activity (the presence of at least one relapse or a new/enlarging T2-FLAIR or gadolinium- enhancing lesion), and its interaction with time adjusted by age, sex, use of disease-modifying therapies and steroids, and prior optic neuritis. The increased rate of neuro-axonal injury during the first five years after onset was more prominent among active patients, as reflected by the changes in GCIPL thickness (p = 0.02), whole brain (p = 0.002) and thalamic volumes (p < 0.001). Thereafter, rates of retinal and brain changes stabilized and were similar in active and stable patients. Focal inflammatory activity is associated with neurodegeneration early in MS which reinforces the use of an early intensive anti-inflammatory therapy to prevent neurodegeneration in MS.

## Introduction

Multiple sclerosis (MS) is an immune-mediated inflammatory and neurodegenerative disorder of the central nervous system (CNS). In MS, neuro-axonal loss is the major substrate of permanent clinical disability^1^ and neuropathological studies indicate it is an early disease phenomenon^2^. In recent years, new algorithms to quantify brain volume and retinal thicknesses have been developed using magnetic resonance imaging (MRI)^3^ and optical coherence tomography (OCT)^4^, fostering more intense research into the dynamics of neuro-axonal injury. Indeed, we recently reported that brain volume loss was more prominent during the first years of the disease^5^.

Beyond disease dynamics, understanding the drivers of neuro-axonal injury and in particular, the role of focal inflammation, a treatable feature of the disease, is paramount to the design of therapeutic strategies. In contrast to the widely accepted two-stage theory, whereby MS is firstly an inflammatory and only subsequently a neurodegenerative condition^6^, current data endorses inflammation as a key driver of neurodegeneration in MS^7–9^. Accordingly, we set out to quantify the impact of focal inflammatory activity on the rate of retinal and brain neuro-axonal damage over the course of MS in the 161 out of the 171 patients of the ongoing MS-VisualPath prospective cohort who fulfilled eligibility criteria using all available visits up to the fifth year of follow-up.

## Methods

### Study population

The ethics committee at the Hospital Clinic of Barcelona approved the study, and written informed consent was obtained from the patients before their inclusion in accordance with the Helsinki Declaration and the study was performed following relevant guidelines and regulations.

The first 171 consecutive patients with MS (according to McDonalds criteria^10–12^) who were enrolled in the open prospective Barcelona MS-Visualpath cohort at the Hospital Clinic-University of Barcelona between February 2011 and February 2019 were evaluated for eligibility^13^. The presence of any neurological disorder other than MS or an ocular pathology, including severe refractive errors (± 6 or a stronger prescription) or a cataract significant enough to affect OCT quality, were exclusion criteria for the Barcelona MS-Visualpath cohort. In our cohort, patients undergo an annual neurological, ophthalmological and MRI examination for up to 3 years, and biannually thereafter.

In this study, we considered the patient data from baseline to the fifth year of follow-up. Out of the 171 patients included in the cohort, 13 and 15 patients were excluded from this study due to: a disease duration longer than 30 years (MRI = 1, OCT = 1), loss of follow-up at baseline (MRI = 5, OCT = 3), not reaching the first-year follow-up (MRI = 6, OCT = 6), recent corticosteroid administration or pregnancy (MRI = 2, OCT = 0), refusal to undergo more than 1 MRI (MRI = 1) or bilateral retinal problems (OCT = 3). Finally, 161 participants representing 94% of the 171 available participants at the data lock date (30th July 2018) were included for OCT models (n = 158) and MRI models (n = 156). As the Barcelona MS-Visualpath cohort is an open cohort, the recruitment is still open and therefore, not all the patients included in the study had 5 years of follow-up at the data lock date. Specifically, we included 156, 139, 122 and 94 patients with 1-year, 2-years 3-years and 5-years of follow-up for MRI analysis and 158, 141, 127, and 100 patients with 1-year, 2-years 3-years and 5-years of follow-up for OCT analysis. The main reason for not being included during the follow-up assessments was not reaching the time point (OCT 11/158 and MRI 13/156 were missing at year 2; OCT 18/158 and MRI 19/156 at year 3; OCT 29/158 and MRI 28/156 at year 5). Other reasons were lack of willingness to continue in the study (OCT: 6/158 patients and MRI: 10/156 patients), time constrictions to comply with protocol (OCT 4/158 and MRI 8/156) and moving to a different city (OCT 9/158 and MRI 9/156). A flowchart defining how the participants in this study were selected is shown in Figure e 1. We included 1,249 OCT scans [median 8 scans/patient with an interquartile range [(IQR) of 6–10 scans and median inter-scan period of 377.5 days, IQR (359–478.5)] and 649 MRI scans [median 4 MRI scans/patient, IQR (3–5) and median inter-scan period of 369 days, IQR (357–399)].

This article follows the STROBE guidelines (see Supplementary materials)^14^ and APOSTEL recommendations^15^.

### Retinal imaging acquisition and processing

Retinal scans were performed on Spectralis spectral-domain OCT devices (Heyex 5.30 Heidelberg Engineering, Heidelberg, Germany) by a trained optometrist (SAA), under standard ambient light conditions (80–100 foot-candles) and using eye-tracking modality without pupillary dilatation. Correction for spherical errors was adjusted prior to each measurement. The technician performing the OCT scans was blind to the patient’s clinical information, and the peripapillary retinal nerve fiber layer (pRNFL) and macular protocol used are explained in the Supplementary materials. We estimated the percentage change in the pRNFL and ganglion cell plus inner plexiform layer (GCIPL) thickness from baseline. All OCT scans fulfilled OSCAR-IB criteria^16^ and any scans with insufficient signal to noise ratios, or retinal thickness algorithm failure, were repeated or the data was excluded (Figure e1).

### Brain imaging acquisition and processing

MRI images were acquired using 32-channel phased-array head coil 3 T Magnetom Trio scanner (Siemens), replaced by a Magnetom Prisma scanner (Siemens) from the 15th January 2018. The acquisition protocol was similar for both scanners (Table e 1). To quantify brain volumes, we registered T2-fluid-attenuated inversion recovery (FLAIR) images to T1-magnetisation prepared rapid acquisition gradient echo (MPRAGE) scans to ease manual segmentation of the lesions by a trained neurologist (IPV). After lesion in-painting of the T1-MPRAGE scan, we applied a registration-based Jacobian integration algorithm to quantify annual changes in whole brain, grey matter (this metric included cortical and deep gray matter) and thalamic volumes relative to the baseline, according to the methodology described elsewhere^5,17^. In addition, a trained neuro-radiologist quantified the number of gadolinium-enhancing T1 lesions (Gad+) and new/enlarging T2-FLAIR lesions.

**Table 1:** Baseline demographic and clinical characteristics of the patients.

	Study population N: 161
Female, n (%)	113 (70.2)
Age, years	40.47 [33.61–48.75]
White-Caucasian, n (%)	160 (99.4)
Disease duration at baseline, years	6.99 [2.84–12.42]
Expanded disability status scale, steps	1.5 [1–2]
Annualized relapse rate (2 years pre inclusion)	0 [0–1]
**Disease type, n (%)**	
Clinically isolated syndrome (CIS)	7 (4.4)
Relapsing remitting multiple sclerosis (RRMS)	139 (86.3)
Secondary progressive multiple sclerosis (SPMS)	5 (3.1)
Primary progressive multiple sclerosis (PPMS)	10 (6.2)
**Disease modifying drugs, n (%)**	
None	38 (23.6)
Interferon beta 1b, subcutaneous	27 (16.8)
Interferon beta 1a, subcutaneous	30 (18.6)
Interferon beta 1a, intramuscular	13 (8.1)
Glatiramer acetate	28 (17.4)
Natalizumab	11 (6.8)
Others^a^	14 (8.8)
**Retinal layer thickness (mean of both eyes), μm (n = 158)**	
Ganglion cell plus inner plexiform layer (GCIPL)	69.2 [62.1–75]
Peripapillary retinal nerve fiber layer (pRNFL)	92.5 [83–102]
Optic neuritis (ON) before the inclusion, n (%)	47 (29.7)
**Brain volumes, cm**^**3**^** (n = 156)**	
Whole brain (parenchymal)^b^	1,540 [1,463–1,598]
Gray matter^b^	802 [763–838]
Thalamus^c^	15.4 [14.6–16.0]
Lesion volume load^d^	5.5 [2.9–11.9]

The data represent median and interquartile range [IQR] for quantitative variables, and the absolute numbers and proportions (%) for the qualitative variables.

^a^Others: teriflunomide (n: 3), dimethyl-fumarate (n: 5), fingolimod (n: 3), rituximab (n: 2), diazoxide (n: 1).

^b^Estimated by SIENA-X, structural image evaluation, using normalisation, of atrophy–cross-sectional.

^c^Estimated by FIRST, FMRIB’s integrated registration and segmentation tool

^d^Voxel count × voxel volume.

### Statistical analyses

We used mixed-effect models with random intercepts and splines to explore non-linear patterns of longitudinal changes in brain volume loss and retinal thinning over time. Third-order B-splines represent a robust statistical approach to explore non-linear relationships in regression, without a priori specification of the non-linear relationship (for instance, a quadratic or cubic assumption)^5^. Although robust, the results are difficult to interpret, relying mostly on graphical representations. Therefore, we used sequential linear splines when a non-linear association was first supported by means of comparison between Akaike Information Criterion (AIC)^18^, resulting from third-order B-splines and linear models. This two-step strategy allowed to maintain the robustness of B-splines as a method to evaluate non-linear association and the interpretability of the parameters derived from the model using linear splines. Results from models based on linear splines including coefficients, graphical representation and predictions could ease the translation of meaningful information to the MS specialists as a first step to bridge the research in MS imaging and MS clinical practice. Using visual inspection of patterns of third-order B-splines together with prior evidence^5^, we selected a single knot at 5 years of disease duration to model our data. These models provided two parameters, beta coefficients, for the change in the rate of annual neuro-axonal loss as the disease progressed. The first parameter provided information about the annual change in the rate of brain volume loss or retinal thinning over the first 5 years and the second, about the change per year after 5 years of disease progression.

Since we hypothesized that inflammatory activity had a differential impact on the rate of neuro-axonal damage throughout the MS disease course, in the models we included inflammatory activity together with its interaction with time (see Supplementary Information for an explanation of the interpretation of the models). Inflammatory activity (active MS) was defined as the presence of either ≥ 1 relapse OR ≥ 1 new/enlarging T2-FLAIR lesion OR ≥ 1 Gad+ lesion in the period of evaluation^19^. We classified MS as stable when none of these attributes existed. By using linear spline mixed models, we finally estimated the predicted rate of brain volume loss or retinal thinning in different case scenarios, defined by the presence or absence of focal inflammatory activity and disease duration. We predicted values for patients with 1, 2, 3, 4, 5, 10 and 15 years of disease duration with and without focal inflammatory activity in the previous year.

Additionally, we included the following fixed effects: sex, age at MS onset (continuous), previous history of optic neuritis (ON, binary), the use of disease-modifying drugs (DMDs, categorical) and steroids (binary) during the period in which neuro-axonal loss was observed (time-varying). We did not include prior history of ON in the whole and gray-matter brain models because we assumed no relevant effect in these areas. However, we included this fixed effect for the retinal and thalamic models, assuming a potential effect of Wallerian and trans-synaptic degeneration, respectively. We only included steroid use in the brain models as current evidence does not support a meaningful effect of steroids on permanent retinal thinning after acute ON. However, we included this fixed variable as a sensitivity analysis to test this assumption. The use of DMDs was modeled as a categorical variable including null, low-intermediate (beta-interferon, glatiramer acetate, teriflunomide, dimethyl fumarate and fingolimod) and high potency (natalizumab, rituximab and alemtuzumab).

We performed four sensitivity analyses: (1) excluding data associated with a disease duration longer than 20 years; (2) excluding data from patients with progressive disease phenotypes; (3) including steroids as a fixed effect in retinal models and (4) excluding data after MRI scanner upgrade.

We estimated 95% percentile confidence intervals (95% CI) for the beta coefficients and the predictions of the rate of brain volume loss or retinal thinning using a parametric bootstrap. The p-values were estimated following Satterthwaite's method^20^. All statistical analyses were performed using R language (version 3.5.0) and the data was analyzed from December 2017 to April 2019.

## Results

### Study population

The study population included 161 patients with MS (70.2% women) with a median age 40.56 years and a median 7.06 years disease duration. Most patients had a relapsing–remitting MS (RRMS) course with mild disability (median expanded disability status scale score—EDSS: 1.5) and they were receiving DMDs at baseline (Table 1).

During the follow-up [median 4.83 (2.87–5.03)], nine naive patients commenced treatment with low-intermediate potency DMDs, 15 patients were withdrawn from a low-intermediate DMD, with a further 27 patients changing to another low-intermediate potency DMD. In addition, six patients ceased treatment and restarted it again with the same DMD, 8 patients changed from natalizumab to fingolimod due to the detection of John Cunningham virus antibodies, and three patients changed from low-intermediate to high potency DMD due to a lack of efficacy.

Furthermore, 107 (66.46%) patients had at least one period with focal inflammatory activity (figure e-2). Among these, 64 patients (39.8%) had at least one relapse (52/64 received steroid), 76 (47.2%) had at least one new T2-FLAIR and in 35 (21.7%) Gad+ lesions appeared. In addition, 6 out of 7 patients progressed from clinically isolated syndrome (CIS) to RRMS, and 2 out of 139 from RRMS to secondary progressive MS (SPMS). There were 13 patients who experienced a unilateral episode of acute ON and one patient had two independent episodes of acute ON in each eye. The retinal scans after these acute episodes were not considered in the analyses.

### Rates of retinal thinning and brain volume loss in terms of inflammatory activity and MS stage

**Figure 1:**
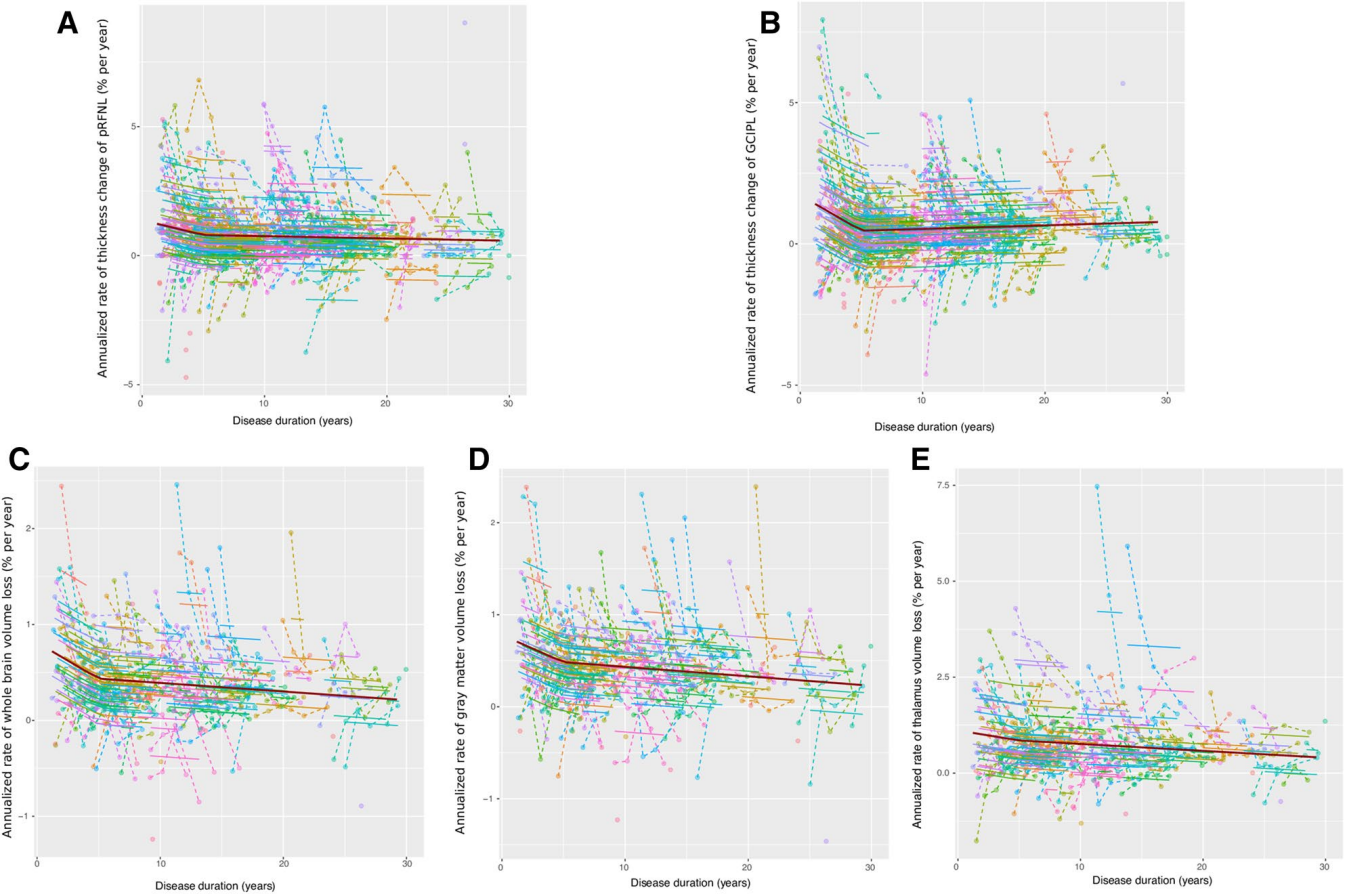
Models of the change in retinal layer thicknesses and brain volume during MS course. The black points joined by dashed lines represent the individual trajectories of the changes in retinal thickness (per eye) or brain volume (per subject), while the thicker curves represent the individual fit of the model (per eye for the retinal model and per subject for the brain volumes) and the dark red line represents the population model. **(A)** Peripapillary retinal nerve fiber layer (pRNFL); **(B)** ganglion cells plus inner plexiform layer (GCIPL); **(C)** whole brain volume; **(D)** gray matter volume; and **(E)** thalamus volume. The Y axis represents the relative change ([visit minus baseline]/baseline) in the eye or brain region. The X axis represents the time (years) from clinical onset. All models are linear spline mixed-effects models following the equation: $\widehat{y } \sim lspline\left(disease duration, knot=5\right)+(1\left|participant+1\right|eye:participant)$ for the retina, and $\widehat{y } \sim lspline\left(disease duration, knot=5\right)+(1|participant)$ for the brain. All the models were fitted using the “lme4” package for R.

According to the AIC, third-order B-Spline mixed-effect models best fitted the distribution of annual changes of retinal thicknesses [AIC linear vs. B-spline models: 2,803 vs. 2,797 (pRNFL); 2,680 vs. 2,659 (GCIPL)]. The annual rates of retinal thinning were only mildly more pronounced for the pRNFL during the first years of the disease (Figure e-3A), yet they increased markedly for GCIPL (Figure e-3B). When we expanded the analyses of the changes in brain volume relative to our previous study^5^, including 16 new subjects and the fifth year of follow-up (94 subjects with a 5 year follow-up), the data supported an accelerated whole brain, gray matter and thalamus volume loss in the first years of the disease, as found previously (Figure e-3 C-E). Prediction from models with Linear-Splines using a single knot at five years of disease duration (Fig. 1) was similar to those with B-splines (Figure e-3). As discussed previously, we based our results on the linear models.

**Figure 2:**
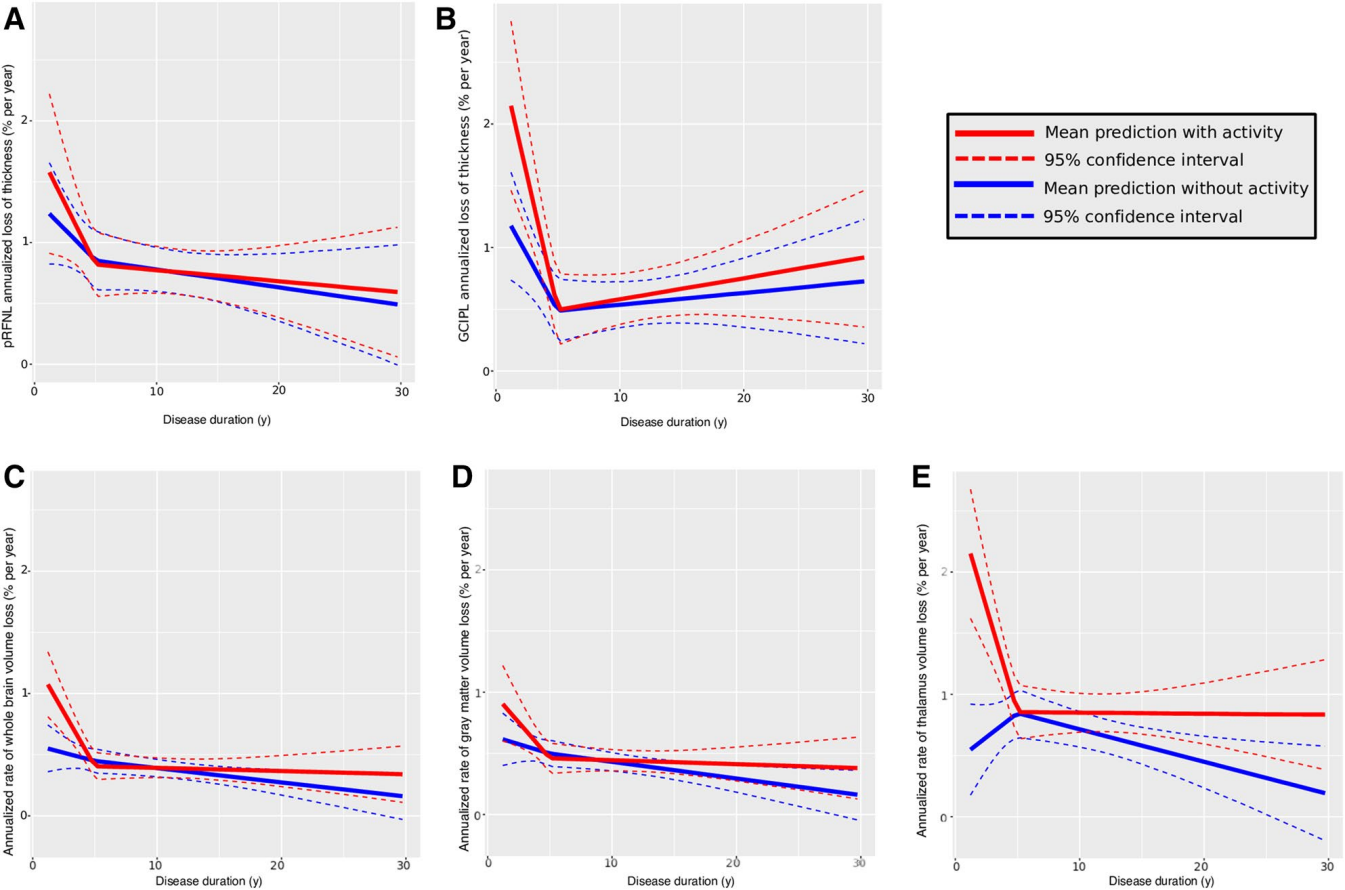
Models of the change in retinal layer thicknesses and brain volume during MS course provoked by focal inflammatory activity. Red represents the prediction with focal inflammatory activity and blue, the prediction in the absence of measurable focal inflammatory activity. The dotted lines represent the 95% confident interval calculated by parametric bootstraps. **(A)** Peripapillary retinal nerve fiber layer (pRNFL); **(B)** ganglion cells plus inner plexiform layer (GCIPL); **(C)** whole brain volume; **(D)** gray matter volume and **(E)** thalamus volume. The Y axis represents the predicted relative change ([visit minus baseline]/baseline) in the eye or brain region. The X axis represents the time (years) from clinical onset. All models are linear spline mixed-effects models following the equation: $\widehat{y } \sim activity*lspline\left(disease duration, knot=5\right)+(1\left|participant+1\right|eye:participant)$ for the retina, and $\widehat{y } \sim activity*lspline\left(disease duration, knot=5\right)+(1|participant)$ for the brain. All the models were fitted using the “lme4” package for R.

MS patients displayed a higher rate of retinal thinning and brain volume loss in the first 5 years of the disease (Fig. 1), particularly regarding the changes in GCIPL thickness (p = 0.02), or whole brain (p = 0.002) and thalamus volumes (p < 0.001) in active patients: Table 2 and Fig. 2. Thereafter, the rates of retinal thinning and brain volume loss stabilized, and they were similar in active and stable patients (as defined by the study protocol: Fig. 2).

**Table 2:** Effect of demographic and MS-related features on the pattern of change in retinal and brain parameters.

Parameters	pRNFL β (95% CI) p-value	GCIPL β (95% CI) p-value	Whole brain β (95% CI) p-value	Gray matter β (95% CI) p-value	Thalamus β (95% CI) p-value
Intercept *β*_*0*_	β: 0.76 (− 0.14, 1.66) p-value: 0.103	β: 1.01 (0.04, 1.97) p-value: 0.043	β: 0.78 (0.40, 1.16) p-value < 0.001	β: 1.11 (0.68, 1.51) p-value < 0.001	β: 0.79 (0.01, 1.55) p-value: 0.04
Focal activity *β*_*1*_	β: 0.43 (− 0.55, 1.42) p-value: 0.386	β: 1.22 (0.21, 2.24) p-value: 0.02	β: 0.68 (0.25, 1.13) p-value: 0.002	β: 0.38 (− 0.12, 0.87) p-value: 0.14	β: 2.11 (1.30, 2.95) p-value: < 0.001
MS duration ≤ 5 years (effect size per year) *β*_*2*_	β: − 0.10 (− 0.22, 0.02) p-value: 0.093	β: − 0.19 (− 0.32, − 0.07) p-value: 0.003	β: − 0.03 (− 0.09, 0.03) p-value: 0.27	β: − 0.04 (− 0.10, 0.03) p-value: 0.26	β: 0.08 (− 0.03, 0.18) p-value: 0.16
MS duration > 5 years (effect size per year) *β*_*3*_	β: − 0.01 (− 0.04, 0.01) p-value: 0.342	β: 0.01 (− 0.02, 0.04) p-value: 0.45	β: − 0.01 (− 0.02, 0.00) p-value: 0.01	β: − 0.02 (− 0.03, − 0.01) p-value: 0.003	β: − 0.03 (− 0.05, − 0.01) p-value: 0.01
Interaction activity and MS duration ≤ 5 years (effect size per year) *β*_*4*_	β: − 0.09 (− 0.30, 0.12) p-value: 0.401	β: − 0.24 (− 0.46, − 0.02) p-value: 0.036	β: -0.15 (-0.24, -0.05) p-value: 0.002	β: − 0.09 (− 0.20, 0.02) p-value: 0.11	β: − 0.43 (− 0.61, − 0.25) p-value: < 0.001
Interaction activity and MS duration > 5 years (effect size per year) *β*_*5*_	β: 0.01 (− 0.02, 0.03) p-value: 0.713	β: 0.01 (− 0.02, 0.04) p-value: 0.654	β: 0.01 (0.00, 0.02) p-value: 0.12	β: 0.01 (0.00, 0.02) p-value: 0.14	β: 0.03 (0.00, 0.05) p-value: 0.02
Sex (male) (reference: female)	β: 0.17 (− 0.17, 0.52) p-value: 0.323	β: 0.25 (− 0.12, 0.62) p-value: 0.188	β: -0.02 (-0.15, 0.12) p-value: 0.82	β: 0.03 (− 0.11, 0.16) p-value: 0.69	β: − 0.13 (− 0.40, 0.14) p-value: 0.35
Age at onset, years	β: 0.01 (− 0.01, 0.03) p-value: 0.191	β: 0.01 (− 0.01, 0.03) p-value: 0.549	β: -0.01 (-0.01, 0.00) p-value: 0.08	β: − 0.01 (− 0.02, − 0.01) p-value: 0.001	β: − 0.01 (− 0.03, 0.00) p-value: 0.08
Low-intermediate potency DMD (reference: none)	β: 0.08 (− 0.19, 0.34) p-value: 0.565	β: 0.23 (− 0.06, 0.50) p-value: 0.111	β: 0.06 (-0.05, 0.17) p-value: 0.28	β: 0.02 (− 0.10, 0.15) p-value: 0.71	β: 0.07 (− 0.15, 0.29) p-value: 0.53
High potency DMD (reference: none)	β: − 0.07 (−0.54, 0.39) p-value: 0.761	β: 0.10 (− 0.41, 0.59) p-value: 0.709	β: 0.14 (-0.09, 0.35) p-value: 0.23	β: 0.07 (− 0.17, 0.32) p-value: 0.55	β: 0.38 (− 0.05, 0.80) p-value: 0.08
Steroid administration (reference: none)	–	–	β: 0.01 (-0.11, 0.13) p-value: 0.86	β: 0.04 (− 0.09, 0.18) p-value: 0.54	β: 0.01 (− 0.22, 0.22) p-value: 0.97
History of optic neuritis (reference: none)	β: 0.31 (0.05,0.56) p-value: 0.02	β: 0.0 (− 0.27, 0.26) p-value: 0.975	–	–	β: 0.19 (− 0.05, 0.43) p-value: 0.12

Data represent beta coefficients, 95% confidence intervals for the annual of retinal and brain change, and the p-values from a linear spline mixed-effects models that include covariates as fixed effects: *pRNFL* peripapillary retinal nerve fiber layer, *GCIPL* ganglion cell plus inner plexiform layer, *DMD* disease modifying drugs.

Table 2 displays the output of the models, including local inflammatory activity, time, its interaction and covariates (Table 2 and Supplementary Information). As indicated, the increase in the rate of brain volume loss and GCIPL thinning stabilized after approximately 5 years, explaining why the β coefficients for the first 5 years (annual change) were negative. Stable MS patients had a higher annualized rate of GCIPL thinning at the beginning of the disease, which then decreased over the following 5 years [β_2_: −0.19%/year 95% CI (− 0.32, − 0.07)], whereas the annualized rate of GCIPL thinning in active patients diminished faster during the same time period [β_2_ + β_4_: (− 0.19) + (− 0.24) = − 0.43%/year 95% CI [(− 0.32 + − 0.46 = − 0.78), (− 0.07 + − 0.02) = − 0.09)]. After 5 years, non-significant slopes for annual rates of GCIPL thinning were found for stable (β_3_: + 0.01%/year) and active patients (β_3_ + β_5_: 0.01 + 0.01%/year) indicating a steady rate of decline over the years. The changes in pRNFL thickness followed a similar trend but they were not estimated to reach statistical significance.

Stable patients did not show a significant decline in the annualized rate of whole brain volume loss during the first five years of disease progression (non-significant β_2_: Table 2), whereas the mean annualized rate of whole brain volume loss declined faster over this period in active patients (whole brain β_2_ + β_4_: mean decline 0.18%/year). After 5 years, similar nearly zero slopes for the rates of brain volume loss were found for stable (whole brain β_3_: − 0.01%/year) and active patients (whole brain β_3_ + β_5_: 0%/year) supporting steady changes after 5 years. Similar trends were found for grey matter and the thalamus (Table 2).

### Impact of early active MS on neurodegeneration

According to the mixed effects linear spline models, the speed of GCIPL thinning and whole brain volume loss was nearly twice as fast in active MS patients than in stable MS patients during the first 2 years after MS onset, while thalamic volume loss was approximately five times faster. These differences disappeared rapidly 5 years after MS onset (for further details see Table 3).

**Table 3:** Predicted rates of annual retinal thinning and brain volume loss in relation to inflammatory activity and time from MS onset.

Years from MS onset	Area	Activity	No activity	Ratio activity/no activity (95% CI)
Year 1	GCIPL	**2.24**	**1.21**	**1.84 (1.16; 3.11)**
	pRNFL	1.26	1.62	1.28 (0.72; 2.14)
	Whole brain	**1.32**	**0.57**	**2.32 (1.48; 4.22)**
	Gray matter	1.10	0.67	1.64 (0.93; 2.98)
	Thalamus	**2.56**	**0.47**	**5.29 (2.06; 32.71)**
Year 2	GCIPL	**1.81**	**1.03**	**1.74 (1.15; 2.75)**
	pRNFL	1.16	1.42	1.22 (0.76; 1.84)
	Whole brain	**0.96**	**0.52**	**1.84 (1.31; 2.68)**
	Gray matter	0.84	0.58	1.44 (0.96; 2.17)
	Thalamus	**1.92**	**0.59**	**3.27 (2.08; 6.58)**
Year 3	GCIPL	**1.37**	**0.85**	**1.61 (1.13; 2.37)**
	pRNFL	1.06	1.22	1.15 (0.81; 1.59)
	Whole brain	**0.70**	**0.49**	**1.43 (1.10; 1.89)**
	Gray matter	0.65	0.53	1.25 (0.91; 1.69)
	Thalamus	**1.46**	**0.68**	**2.15 (1.59; 3.14)**
Year 4	GCIPL	0.67	0.93	1.39 (1.01; 1.98)
	pRNFL	0.96	1.02	1.07 (0.83; 1.36)
	Whole brain	**0.52**	**0.47**	**1.13 (0.86; 1.46)**
	Gray matter	0.53	0.49	1.09 (0.81; 1.48)
	Thalamus	**1.14**	**0.74**	**1.53 (1.18; 2.09)**
Year 5	GCIPL	0.49	0.50	1.01 (0.52; 1.88)
	pRNFL	0.85	0.82	0.96 (0.70; 1.29)
	Whole brain	0.42	0.45	0.94 (0.69; 1.24)
	Gray matter	0.47	0.46	0.98 (0.69; 1.35)
	Thalamus	0.94	0.79	1.2 (0.90; 1.61)
Year 10	GCIPL	0.54	0.58	1.09 (0.77; 1.52)
	pRNFL	0.79	0.70	0.99 (0.80; 1.22)
	Whole brain	0.44	0.43	1.04 (0.80; 1.32)
	Gray matter	0.48	0.48	1.01 (0.77; 1.31)
	Thalamus	0.84	0.77	1.14 (0.89; 1.46)
Year 15	GCIPL	0.59	0.583	1.15 (0.84; 1.59)
	pRNFL	0.71	0.73	1.03 (0.79; 1.32)
	Whole brain	0.42	0.32	1.33 (0.97; 1.86)
	Gray matter	0.47	0.39	1.20 (0.87; 1.65)
	Thalamus	0.88	0.58	1.51 (0.13; 2.15)

The data represents the predicted annualized rates (%/year) of retinal thinning and brain volume loss using linear splines mixed effect models. In addition to estimates, we added ratios to ease interpretation. The 95% confident intervals were calculated by parametric bootstraps and significant differences are indicated in bold.

### Effect of demographic and MS-related characteristics on the dynamics of the changes in retinal thickness and brain volume

We did not find any significant association between any of the changes in retinal layer thickness and sex, age at MS onset, or DMD use. The presence of prior ON was only significantly associated with pRNFL [β: 0.31 95% CI (0.05, 0.56); p-value: 0.02]. However, as described previously, we found a significant association between age at MS onset and the gray matter volume change [β: − 0.01 95% CI (− 0.02, − 0.01); p-value: 0.001], and similar trends for the whole brain and thalamus (Table 2). Nevertheless, the magnitude of the effect was relatively weak compared to that of inflammation [annualized rate of gray matter volume loss 0.1% per decade of delay in the age of MS onset].

### Sensitivity analyses

Similar findings were found after excluding the data with a disease duration longer than 20 years (Figure e-4) and that from progressive disease subtypes (Figure e-5). The retinal changes were similar in models that included steroids as a fixed effect, with no relevant changes (Table e-2). Similar findings were found after excluding the data obtained after the MRI upgrade (Figure e-6).

## Discussion

The main findings of this study were two-fold: (1) there was an accelerated rate of retinal thinning and brain volume loss during the first 5 years after the clinical onset of MS, mainly driven by focal inflammatory activity; and (2) the dynamics of the changes in the retina and brain were similar, and similarly related to focal inflammatory activity.

We previously described an increased rate of brain volume changes in early MS^5^ and here, we provide further evidence of the key role of focal inflammatory activity in this early accelerated loss in brain volume. The effect of inflammatory activity was more prominent in the thalamus than in the cortical gray matter, consistent with recent studies suggesting that the thalamus is one of the first atrophic regions in MS, later followed by regions of cortical gray matter^21^. Focal brain lesions may cause neuro-axonal loss due to axonal transection^22^ and hence, the sensitivity of the thalamus to early volume changes in active MS may be explained by its profile as a connectivity hub. Damage to thalamic projecting fibers could provoke a loss of the thalamic neuropil and consequently, thalamic volume loss. The potential effect of *pseudoatrophy* must also be considered when assessing brain volume loss, involving the resolution of on-going inflammation at the initiation of DMD or steroid therapy. Yet while we cannot completely rule this out, our findings suggest this would only have had a marginal effect here. First, patients were recruited after 1 month without a relapse or on steroid therapy, and the annual examinations were performed under stable conditions even though this might imply delaying MRI acquisition for patients receiving steroids due to a relapse during the follow-up. Second, DMD or steroid use did not appear to significantly affect brain volume loss. Third, the rate of GCIPL thinning was similar, a layer not affected by inflammatory changes^23^.

The similarity in the dynamics of retinal and brain changes, and their relationship to focal inflammation indicates that retinal changes mirror brain changes in MS, and that they may therefore reflect the global CNS disease burden. The mechanisms by which the retina is damaged in MS may involve primary retinal degeneration^24^, trans-synaptic degeneration due to lesions in the posterior afferent visual pathway^23^ and retrograde degeneration due to inflammation (clinical or sub-clinical) in the optic nerve^25^. The close time relationship observed in our study between focal brain inflammatory activity and retinal changes suggests a fundamental role of sub-clinical micro-inflammation in the optic nerve for retinal neuro-axonal damage in the first years of MS onset. MRI inflammatory activity was previously found to affect GCIPL thinning throughout the course of MS in patients followed over 21 months^26^. By contrast, here the effect of focal inflammatory activity on GCIPL thinning was notable during the first 5 years but it declined rapidly thereafter. Moreover, changes in the GCIPL were more prominent than in the pRNFL and likewise, the effects differed depending on disease activity, as suggested previously^26^. If sub-clinical micro-inflammation in the optic nerve is the causal link, pRNFL thinning may be underestimated due to the presence of swelling. Alternatively, the soma of macular ganglion cells and their dendritic branches may be more sensitive to damage than retinal axons.

Our findings may have practical implications for MS clinical management and drug development. Based on our results, the first 5 years of MS represents the optimal therapeutic window to protect the CNS^27^. Indeed, the earlier inflammatory activity is controlled, the greater the benefits expected in terms of neurodegeneration. Our findings reinforce the idea that long-term outcomes are more favorable following early intensive therapy as opposed to the first-line use of moderate-efficacy DMDs^28^. Although the rates of brain and retinal changes diminish after the first 5 years, they do not reach zero, and similar trends were observed for active and stable MS patients (except regarding thalamic volume loss). These findings may indicate that mechanisms other than acute inflammation may take over to maintain a steady rate of neuro-axonal damage. While an adaptive immune response against the CNS seems to be the main contributor to focal inflammatory lesions, compartmentalized immune reactions supported by the innate immune system are likely to drive late neuroinflammation and degeneration^29^. Both the adaptive and innate immune systems are important over the entire course of MS and they can explain the full phenotypic disease spectrum, as revealed by computational simulations^8^. However, their differential contribution to early and late MS may offer a biological explanation for our findings, suggesting alternative therapeutic targets for future drug development, such as key innate immune system mediators. Indeed, Ibudilast targets neuroinflammatory mediators and it has a positive effect on brain volume loss, as recently reported in a phase II trial for PMS^30^.

This study has several strengths. First, we evaluated the effects of local inflammatory activity on neuro-axonal damage using retinal and brain measurements, in order to obtain evidence that the interplay between focal inflammatory activity and neuro-axonal damage in MS is a global and consistent phenomenon in the CNS. Using OCT and MRI evens out the limitations of each of these imaging techniques (e.g. the influence of pseudoatrophy in MRI or of quantifying a small region of the CNS in retinal OCT). Second, we evaluated the goodness of fit of linear and non-linear models without the a priori assumption of linearity. Third, the quantification of the retinal and brain changes was performed blind to MS-related characteristics. Finally, we used mixed-effect models as a robust technique to analyze repeated measurements, with missing data assumed to be missing at random.

Our study also has some limitations. First, the cohort was too small to perform subgroup analyses by MS and/or DMD type. A much larger sample would have been needed and probably, other methods to deal with time-varying treatments and time-dependent confounders. Second, we have no data regarding the genetic or immune profiles of the patients limiting our ability to assess the biological pathways underlying our findings. Finally, our study was underpowered to interrogate the effects of different levels of disease activity.

The relationship between inflammation and neurodegeneration in MS is complex. In our study, we aimed to assess the immediate or short-term impact of ongoing focal inflammatory activity on the rates of retinal thinning and brain volume loss. Further investigations should address the effect of accumulated inflammatory load and the possibility of long-lasting effects of ongoing focal inflammatory activity due to other potential mechanisms (e.g. trans-synaptic degeneration).

In conclusion, there is an accelerated rate of brain and retinal neuro-axonal damage in the early stages of the MS disease course driven by focal inflammatory activity. Consequently, a beneficial neuroprotective strategy should focus on halting inflammatory activity during this optimal treatment window. As the magnitude of the effect of focal inflammatory activity on neuro-axonal damage declines over time, other mechanisms like compartmentalized inflammation may become more important, conferring a challenge to clinical management but also, a novel therapeutic opportunity that might be explored for late MS.

## Supplementary information

Supplementary Information.

## Data Availability

Anonymized data will be shared by request from any qualified investigator for the purpose of replicating the results presented, provided that data transfer follows the EU legislation on general data protection.

## Abbreviations

95% CI: 95% confidence interval
AIC: Akaike information criterion
CIS: Clinically isolated syndrome
CNS: Central nervous system
DMD: Disease-modifying drug
FLAIR: Fluid-attenuated inversion recovery
Gad+: Gadolinium enhancing lesions
GCIPL: Ganglion cell plus inner plexiform layer
MPRAGE: Magnetisation prepared rapid acquisition gradient echo
MRI: Magnetic resonance imaging
MS: Multiple sclerosis
OCT: Optical coherence tomography
pRNFL: Peripapillary retinal nerve fiber layer
RRMS: Relapsing remitting multiple sclerosis
SPMS: Secondary progressive multiple sclerosis
