# Supplementary material for: Retinal and brain damage during multiple sclerosis course: inflammatory activity is a key factor in the first 5 years
Source: Sci Rep. 2020 Aug 7;10:13333. doi: 10.1038/s41598-020-70255-z (PMC7414206; doi:10.1038/s41598-020-70255-z)
Supplement: Supplementary file 1 — Supplementary Information. [file 41598_2020_70255_MOESM1_ESM.docx]

**Supplementary material**

**Retinal and brain damage during multiple sclerosis course: inflammatory activity is a key factor in the first five years**

Irene Pulido-Valdeolivas^1^ MD, PhD; Magí Andorrà^1^, MSc; David Gómez-Andrés^2^ MD, PhD; Kunio Nakamura^3^, PhD; Salut Alba-Arbalat^1^, BSc; Erika J. Lampert^4^, BSc; Irati Zubizarreta^1^, MD PhD; Sara Llufriu^1^, MD, PhD; Eloy Martinez-Heras^1^, PhD; Elisabeth Solana^1^, PhD; Nuria Sola-Valls^1^, MD; María Sepulveda^1^, MD; Ana Tercero-Uribe^1^, MD; Yolanda Blanco^1^, MD, PhD; Anna Camos-Carreras^5^ MD; Bernardo Sanchez-Dalmau^5^, MD PhD; Pablo Villoslada^1^, MD, PhD; Albert Saiz^1^, MD, PhD; Elena H. Martinez-Lapiscina^1^* MD, MPH, PhD

^1^ Center of Neuroimmunology and Service of Neurology, Hospital Clinic of Barcelona, Institut d’Investigacions Biomèdiques August Pi Sunyer (IDIBAPS), University of Barcelona, Villarroel 170, Barcelona ES 08036, Spain.

^2^ Child Neurology Unit, Hospital Universitari Vall d'Hebron, Vall d'Hebron Research Institute (VHIR), EURO-NMD and RND-ERN, Passeig de la Vall d’Hebron 119-129, ES 08035, Barcelona, Spain.

^3^ Department of Biomedical Engineering, Lerner Research Institute, Cleveland Clinic, 9500 Euclid Avenue, Cleveland Ohio, 44195, USA.

^4^ Cleveland Clinic Lerner College of Medicine, 9500 Euclid Avenue, Cleveland Ohio, 44195, USA.

^5^ Service of Ophthalmology, Hospital Clinic of Barcelona, Institut d’Investigacions Biomèdiques August Pi Sunyer (IDIBAPS), University of Barcelona, Villarroel 170, Barcelona ES 08036, Spain.

|  | Pages |
| --- | --- |
|  |  |
| Figure e-1: Flowchart of OCT-MRI data. | 2-3 |
|  |  |
| Protocol for OCT and MRI acquisition. | 4 |
|  |  |
| Table e-1: MRI acquisition protocol for both scanners. | 5 |
|  |  |
| Explanation of the model interpretation. | 6-8 |
|  |  |
| Figure e-2: Heatmap showing focal inflammatory activity in every patient at all the study visits. | 9 |
|  |  |
| Figure e-3: Dynamics of annualized rate (%/year) of pRNFL (A) and GCIPL (B) thinning, and whole brain (D), gray matter (E) and thalamus (F) volume loss **in function of disease duration** | 10 |
|  |  |
| Figure e-4: Sensibility analysis excluding data with more than 20 years evolution. | 11 |
|  |  |
| Figure e-5: Sensibility analysis excluding patients with progressive forms. | 12 |
|  |  |
| Figure e-6: Sensitivity analysis including (continuous line) and excluding (dashed lines) data obtained after the MRI upgrade | 13 |
|  |  |
| Table e-2: Comparing the models of the dynamics of annualized rate of the loss in pRNFL and GCIPL thickness in which steroid administration was considered as a fixed effect or not. | 14 |
|  |  |
| Strobe list | 15-16 |

**Figure e-1. Flowchart of OCT-MRI data.**

**OCT:** Flowchart of the OCT data. The boxes on the left refer to OCT exclusions not subject to exclusion because they affect only one eye or one time-point. Retinal alterations included: retinoschisis, pigment epithelium detachment, macular microcysts or macular scar. The boxes on the right refer to subject exclusion. Specific reasons for the missing data during the follow-up are indicated in the flowchart using superscripts: a) 1 lack of willingness;; 1 not enough time; 1 moved to another city; b) 1 not enough time; 2 moved to another city; c) 2 lack of willingness; 0 not enough time; 3 moved to another city; d) 4 lack of willingness; 2 not enough time; 5 moved to another city.

**MRI:** Flowchart of the MRI data. The boxes on the left refer to the MRI exclusions not involving subject exclusion. The boxes on the right refer to subject exclusions. Specific reasons for the missing data during the follow-up are indicated in the flowchart using superscripts: a) 1 lack of willingness; 3 not enough time; 1 moved to another city; b) 0 lack of willingness; 2 not enough time; 1 moved to another city; c) 4 lack of willingness; 1 not enough time; 4 moved to another city; d) 6 lack of willingness; 5 not enough time; 4 moved to another city

**Protocol for OCT and MRI acquisition**

**Optical coherence tomography protocol**

OCT was performed under standard ambient light conditions (80–100 foot-candles) and using eye-tracking modality without pupillary dilatation. Correction for spherical errors was adjusted prior to each measurement. The technician performing the OCT scans was blind to the patient’s clinical information.

The peripapillary retinal nerve fiber layer (pRNFL) thickness was measured using a ring scan of 12 degrees diameter automatically centred on the optic nerve head [100 ART; 1,536 A-Scans per B scan]. The macular scan protocol included a 20x20 degree horizontal raster scan centred on the fovea, including 25 B scans [ART≥9; 512 A-Scans per B scan]. The same optometrist (SAA) performed the intra-retinal layer segmentation to quantify the macular ganglion cell plus inner plexiform layer (GCIPL) thickness in the 6 mm ring area using the same standard 6.0c version of the Spectralis segmentation algorithm in a semi-automated fashion, with manual correction of obvious errors.

**Brain imaging acquisition protocol**

For both scanners, we used the 3-dimensional (3-D) structural T1-weighted magnetization-prepared rapid gradient echo (T1-MPRAGE: voxel size 0.9 x 0.9 x 0.9 mm^3^), 3-D T2-fluid-attenuated inversion recovery (T2-FLAIR) images with the same voxel size and gradient-echo T1 axial images (voxel size 0.7 x 0.6 x 3.0 mm^3^) after gadolinium infusion.

**Brain imaging preprocessing**

We registered T2-fluid-attenuated inversion recovery (FLAIR) images to T1-magnetisation prepared rapid acquisition gradient echo (MPRAGE) scans to ease manual segmentation of the lesions by a trained neurologist (IPV).

**Table e-1. MRI acquisition protocol for both scanners.**

| **Protocol Main Features** | **Siemens Trio** | **Siemens Prisma T2** |
| --- | --- | --- |
|  | **MPRAGE** | |
| Slices [n] | 208 | 208 |
| FoV [mm] | 220 | 220 |
| Slice Thickness [mm] | 0,86 | 0,86 |
| TR [ms] | 1970 | 1970 |
| TE [ms] | 2,41 | 2,51 |
| TI [ms] | 1050 | 1050 |
| Echo Spacing [ms] | 7,2 | 7 |
| Flip Angle [º] | 9 | 9 |
| Fat Supression [Yes/No] | No | No |
| Water Supression [Yes/No] | No | No |
| Matrix Size [pixels/slice] | 256x256 | 256x256 |
| Acceleration | GRAPPA | GRAPPA |
| Acceleration Factor | 2 | 2 |
| Coil Channels [n] | 32 | 32 |
|  | **FLAIR** | |
| Slices [n] | 208 | 208 |
| FoV [mm] | 220 | 220 |
| Slice Thickness [mm] | 0.86 | 0.86 |
| TR [ms] | 5000 | 5000 |
| TE [ms] | 393 | 303 |
| TI [ms] | 1800 | 1800 |
| Echo Spacing [ms] | 3.46 | 3.7 |
| Flip Angle [º] | - | - |
| Fat Supression [Yes/No] | No | No |
| Water Supression [Yes/No] | No | No |
| Matrix Size [pixels/slice] | 256x256 | 256x256 |
| Acceleration | GRAPPA | GRAPPA |
| Acceleration Factor | 2 | 2 |
| Coil Channels [n] | 32 | 32 |
|  | **T1-Gadolinium** | |
| Slices [n] | 46 | 46 |
| FoV [mm] | 240 | 240 |
| Slice Thickness [mm] | 3 | 3 |
| TR [ms] | 390 | 390 |
| TE [ms] | 2.65 | 2.65 |
| TI [ms] | - | - |
| Echo Spacing [ms] | - | - |
| Flip Angle [º] | 90 | 90 |
| Fat Supression [Yes/No] | No | No |
| Water Supression [Yes/No] | No | No |
| Matrix Size [pixels/slice] | 384x384 | 384x384 |
| Acceleration | GRAPPA | GRAPPA |
| Acceleration Factor | 2 | 2 |
| Coil Channels [n] | 32 | 32 |

**Explanation of model parameters interpretation**

For each retinal and brain MRI parameter, we established a model including a linear spline with a knot at 5 years for MS duration and the interaction with activity of this linear spline. Moreover, we included several covariables as fixed effects (age at onset, sex, type of disease modifying drug received during the observed period, …)

Let us express the model according to the following equation:

$$\hat{y} \approx\beta_{0}+ \beta_{1}*activity+\vec{\beta}*linear.spline(t, knot=5)+\sum\beta_{covariable}*covariable+(1|random)$$

where $\hat{y}$is the dependent variable,$activity$ is a binary variable with value = 0 in absence of activity and value=1 if activity is present in that period, $t$ indicates MS duration, $\vec{\beta}$ is a vector of coefficients to adjust the linear spline, $\sum\beta_{covariable}*covariable$ indicates that the model is additionally adjusted for other covariables, and $(1|random)$ indicates that we used a random intercept.

Given that ${if t\leq5, t}_{t\leq5}=t and t_{t>5}=0$ and that ${if t> 5, t}_{t\leq5}=5 and t_{t>5}=t-5$, we can expresses the previous equation as:

$$\hat{y} \approx\beta_{0}+ \beta_{1}*activity+ \beta_{2}*t_{t\leq5}+ \beta_{3}* t_{t>5}+ {\beta_{4}*activity* t}_{t\leq5}+ {\beta_{5}*activity* t}_{t>5}+\sum\beta_{covariable}*covariable+(1|random)$$

where $\beta_{0}$ is the intercept, $\beta_{1}$ is the coefficient for activity, $\beta_{2}$ and $\beta_{3}$ are the coefficients for MS duration (by means of the linear spline, we are able to adjust two different slopes for different periods of MS duration: $\beta_{2}$ for MS duration lower or equal to 5 years and $\beta_{3}$ for MS duration higher than 5 years), and $\beta_{4}$ and $\beta_{5}$ are the coefficients for the interaction between activity and MS duration (again, by means of the linear spline, we adjusted two different slopes for different periods of MS duration).

As $\beta_{1}$, $\beta_{2}$, $\beta_{3}$, $\beta_{4}$ and $\beta_{5}$ may not have a simple interpretation, we decided to develop the preceding formula and provide the reader with a graphical explanation of their meanings.

If $t=0$ (MS onset) $\underset{\Rightarrow}{}$

$\hat{y}$ $\approx\beta_{0}+ \beta_{1}*activity$ $+\sum\beta_{covariable}*covariable+(1|random)$ $\underset{\Rightarrow}{}\hat{y}\left( activity=1 \right)= \beta_{0}+ \beta_{1}+\sum\beta_{covariable}*covariable+\left( 1 | random \right)=$ $\beta_{1}+ \hat{y}\left( activity=0 \right)\underset{\Rightarrow}{}\beta_{1}$is how much $\hat{y}$increases in the presence of activity at MS onset (obviously, this is a theoretical value).

If we split the formula for periods in which there is no or some activity, and in which MS duration is less than or equal to 5 years ($t\leq5$) and greater than 5 years ($t>5$), we would obtain four different expressions:

1) If $t\leq5$ and $activity=0 \underset{\Rightarrow}{}\hat{y} \approx\beta_{0}+ \beta_{2}t+\sum\beta_{covariable}*covariable+(1|random)$ $\underset{\Rightarrow}{}\beta_{2}$ indicates how much $\hat{y}$ increases per year in the first 5 years of MS in the absence of activity

2) If $t>5$ and $activity=0 \underset{\Rightarrow}{}\hat{y} \approx\beta_{0}+ \beta_{3}t+\sum\beta_{covariable}*covariable+(1|random)$ $[notice that \beta_{0}\neq{\beta_{0}}^{'}]\underset{\Rightarrow}{}\beta_{3}$ indicates how much $\hat{y}$ increases per year after 5 years of MS in the absence of activity

3) If $t\leq5$ and $activity=1\underset{\Rightarrow}{}\hat{y} \approx(\beta_{0}+ {\beta_{1})+ (\beta}_{2}+\beta_{4})t+\sum\beta_{covariable}*covariable+(1|random) \underset{\Rightarrow}{}\beta_{2}{+ \beta}_{4}$ indicates how much $\hat{y}$ increases per year in the first 5 years of MS in the presence of activity and $\beta_{4}$ is the difference in the increment of $\hat{y}$per year between the presence and absence of inflammation during the first 5 years of MS

4) If $t>5$ and $activity=0 \underset{\Rightarrow}{}\hat{y} \approx(\beta_{0}+\beta_{1})+(\beta_{3}+\beta_{5})t+\sum\beta_{covariable}*covariable+(1|random)$ $\underset{\Rightarrow}{}{\beta_{3}+\beta}_{5}$ indicates how much $\hat{y}$ increases per year after 5 years of MS in the presence of activity and $\beta_{5}$ is the difference in the increment of $\hat{y}$ per year between the presence and absence of inflammation after 5 years of MS

Graphically, the coefficients may be explained as follows:

At the onset of MS, in the absence of activity, the averaged predicted value for the annual rate of brain volume loss or retinal thinning (AR-BVL/RT) would theoretically be $\beta_{0}+\sum\beta_{covariable}*covariable$, which is the sum of the intercept and the value of the additional covariables. In the presence of activity, the averaged AR-BVL/RT at the beginning of the disease would be $\beta_{0}+\sum\beta_{covariable}*covariable+ \beta_{1}$, So, $\beta_{1}$is an indicator of the theoritcal excess of AR-BVL/RT at MS onset attributable to focal inflammatory activity.

During the first five years, in absence of activity the AR-BVL/RT would change at $\beta_{2}$ per year, meaning that the predicted rate would increase $\beta_{2}$% per year from the MS onset until 5 years. In the presence of activity, the change would be $\beta_{2}+ \beta_{4}$ per year. So, $\beta_{4}$ is the difference in the change of AR-BVL/RT attributable to focal inflammatory activity.

After five years, in the absence of activity the AR-BVL/RT would change at $\beta_{3}$ every year of additional MS duration after 5 years. In the presence of activity, the change would increase $\beta_{5}$ per year relative to periods in which there is no activity.

**Figure e-2: Heatmap showing the focal inflammatory activity in every patient at all visits in the study**. The data is shown for the year prior to the visit and the patients are ordered according to the disease duration at inclusion in the cohort.

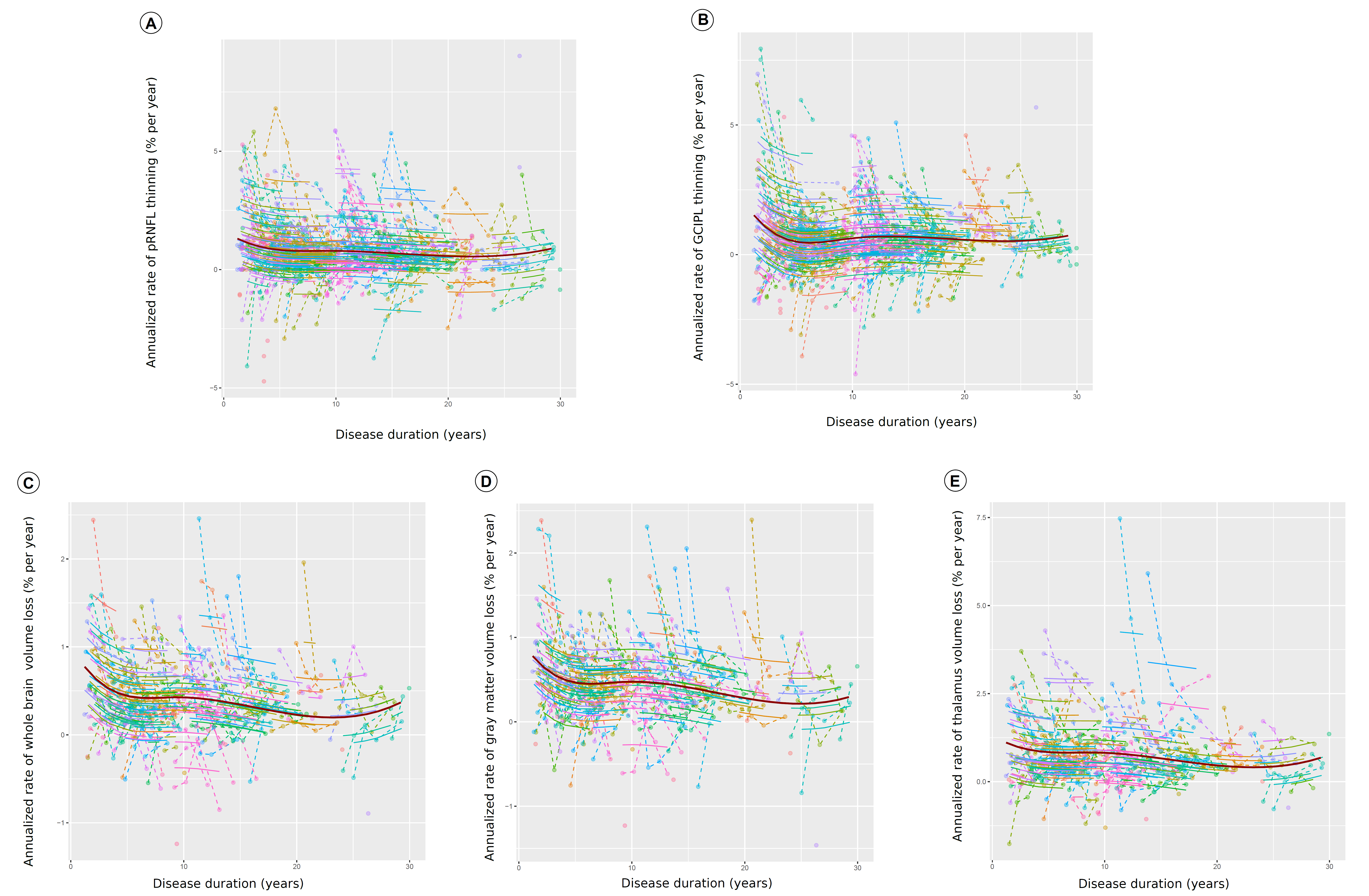


**Figure e-3: Dynamics of the annualized rate (%/year) of pRNFL (A) and GCIPL (B) thinning, and of whole brain (C), gray matter (D) and thalamus (E) volume loss in function of disease duration.** Colored points joined by a line represent the individual trajectories of the loss of thickness for each pair of participants (and eyes in the retinal models). The thicker curves represent the individual fit of the model for that participant (or eye in retinal models). The dark red line shows the population model. Models are bs-spline mixed-effects models following the equation: $\hat{y} \sim Bspline\left( disease duration, knot=median(disease duration) \right)+(1\left| participant+1 \right|eye:participant)$ and $\hat{y} \sim lspline\left( disease duration, knot=median(disease duration) \right)+(1|participant)$for the brain. One knot is at percentile 50 of disease duration.

**Figure e-4: Sensitivity analysis excluding data with more than 20 years evolution** in the dynamics of annualized rate of loss (%/year) for pRNFL thickness (A), GCIPL thickness (B), whole brain volume (C), gray matter volume (D), and thalamus (E) over the disease course as sub-groups of inflammatory activity (no activity, activity).

**Figure e-5: Sensitivity analysis excluding patients with progressive forms** of the dynamics of annualized rate of loss (%/year) in pRNFL thickness (A), GCIPL thickness (B), whole brain volume (C), gray matter volume (D) and thalamus (E) as the disease progresses, as sub-groups of inflammatory activity (no activity, activity).

**Figure e-6: Sensitivity analysis including (continuous line) and excluding (dashed lines) data obtained after the MRI upgrade:** whole brain volume (A), gray matter volume (B) and thalamus (C) as the disease progresses, as sub-groups of inflammatory activity (no activity, activity)

**Table e-2: Comparison of the models of the annualized rate of pRNFL and GCIPL thinning over time before (main manuscript and here in grey) and after including steroid administration as a fixed effect.**

| Covariates | pRNFL β (95% CI)  p-value | | GCIPL β (95% CI)  p-value | |
| --- | --- | --- | --- | --- |
|  | Without steroids | With steroids | Without steroids | With steroids |
| Intercept | β: 0.76 (-0.14,1.66)  p-value: 0.103 | β:0.74(-0.16,1.65)p-value:0.11 | β: 1.01 (0.04,1.97)  p-value: 0.043 | β:1.02 (0.04,1.98)  p-value:0.04 |
| MS duration ≤5 years | β: 0.43 (-0.55,1.42)  p-value: 0.386 | β:-0.10(-0.22,0.02)p-value:0.09 | β: 1.22 (0.21,2.24)  p-value: 0.02 | β:-0.19(-0.32,-0.06)p-value:0.00 |
| MS duration >5 years | β: -0.10 (-0.22,0.02)  p-value: 0.093 | β:-0.01(-0.04,0.01)p-value:0.34 | β: -0.19 (-0.32,-0.07)  p-value: 0.003 | β:0.01(-0.02,0.04)p-value:0.45 |
| Activity | β: -0.01 (-0.04,0.01)  p-value: 0.342 | β:0.48(-0.49,1.46)p-value:0.35 | β: 0.01 (-0.02,0.04)  p-value: 0.45 | β:1.13(0.12,2.16)p-value:0.03 |
| Interaction activity and MS duration ≤5 years | β: -0.09 (-0.30,0.12)  p-value: 0.401 | β:-0.10(-0.31,0.11)  p-value:0.38 | β: -0.24 (-0.46,-0.02)  p-value: 0.036 | β:-0.23(-0.46,-0.01)p-value:0.04 |
| Interaction activity and MS duration >5 years | β: 0.01 (-0.02,0.03)  p-value: 0.713 | β:0.01(-0.02,0.03)p-value:0.69 | β: 0.01 (-0.02,0.04)  p-value: 0.654 | β:0.01(-0.02,0.04)p-value:0.68 |
| Sex (male) | β: 0.17 (-0.17,0.52)  p-value: 0.323 | β:0.17(-0.18,0.52)p-value:0.32 | β: 0.25 (-0.12,0.62)  p-value: 0.188 | β:0.25(-0.12,0.63)p-value:0.19 |
| Age at onset | β: 0.01 (-0.01,0.03)  p-value: 0.191 | β:0.01(-0.01,0.03)p-value:0.19 | β: 0.01 (-0.01,0.03)  p-value: 0.549 | β:0.01(-0.01,0.03)p-value:0.56 |
| DMT level: low-intermediate (reference: none) | β: 0.08 (-0.19,0.34)  p-value: 0.565 | β:0.09 (-0.18,0.35)  p-value:0.52 | β: 0.23 (-0.06,0.50)  p-value: 0.111 | β:0.21(-0.08,0.49)p-value:0.15 |
| DMT level: high (reference: none) | β: -0.07 (-0.54,0.39)  p-value: 0.761 | β:-0.06(-0.53,0.40)p-value:0.79 | β: 0.10 (-0.41,0.59)  p-value: 0.709 | β:0.08(-0.42,0.58)p-value:0.77 |
| Steroid administration | --- | β:-0.07(-0.30,0.17)p-value:0.57 | --- | β:0.14(-0.10,0.39)p-value:0.25 |
| History of Optic Neuritis | β: 0.31 (0.05,0.56)  p-value: 0.02 | β:0.31(0.05,0.56)p-value:0.02 | β: 0.0 (-0.27,0.26)  p-value: 0.975 | β:-0.01(-0.27,0.27)p-value:0.96 |

STROBE Statement—checklist of items that should be included in reports of observational studies

|  | Item No | Man.  Pag. | | Recommendation |
| --- | --- | --- | --- | --- |
| **Title and abstract** | 1 | 3 | | (*a*) Indicate the study’s design with a commonly used term in the title or the abstract |
|  |  | 3 | | (*b*) Provide in the abstract an informative and balanced summary of what was done and what was found |
|  | | | Introduction | |
| Background/rationale | 2 | 4 | | Explain the scientific background and rationale for the investigation being reported |
| Objectives | 3 | 4 | | State specific objectives, including any prespecified hypotheses |
|  | | | Methods | |
| Study design | 4 | 4-5 | | Present key elements of study design early in the paper |
| Setting | 5 | 4-5 | | Describe the setting, locations, and relevant dates, including periods of recruitment, exposure, follow-up, and data collection |
| Participants | 6 | 4-5 | | (*a*) *Cohort study*—Give the eligibility criteria, and the sources and methods of selection of participants. Describe methods of follow-up  *Case-control study*—Give the eligibility criteria, and the sources and methods of case ascertainment and control selection. Give the rationale for the choice of cases and controls  *Cross-sectional study*—Give the eligibility criteria, and the sources and methods of selection of participants |
|  |  |  | | (*b*) *Cohort study*—For matched studies, give matching criteria and number of exposed and unexposed  *Case-control study*—For matched studies, give matching criteria and the number of controls per case |
| Variables | 7 | 5-8 | | Clearly define all outcomes, exposures, predictors, potential confounders, and effect modifiers. Give diagnostic criteria, if applicable |
| Data sources/ measurement | 8* | 5-8 | | For each variable of interest, give sources of data and details of methods of assessment (measurement). Describe comparability of assessment methods if there is more than one group |
| Bias | 9 | 9-11 | | Describe any efforts to address potential sources of bias |
| Study size | 10 | 6-8, figure e1 | | Explain how the study size was arrived at |
| Quantitative variables | 11 | 5-8 | | Explain how quantitative variables were handled in the analyses. If applicable, describe which groupings were chosen and why |
| Statistical methods | 12 | 6-9 | | (*a*) Describe all statistical methods, including those used to control for confounding |
|  |  | 6-9 | | (*b*) Describe any methods used to examine subgroups and interactions |
|  |  | 6-9 | | (*c*) Explain how missing data were addressed |
|  |  | 4-8 | | (*d*) *Cohort study*—If applicable, explain how loss to follow-up was addressed  *Case-control study*—If applicable, explain how matching of cases and controls was addressed  *Cross-sectional study*—If applicable, describe analytical methods taking account of sampling strategy |
|  |  | 11 | | (*e*) Describe any sensitivity analyses |

|  | | | Results | | |
| --- | --- | --- | --- | --- | --- |
| Participants | 13* | 9,10, figure e1 | | | (a) Report numbers of individuals at each stage of study—eg numbers potentially eligible, examined for eligibility, confirmed eligible, included in the study, completing follow-up, and analysed |
|  |  | 10, figure e1 | | (b) Give reasons for non-participation at each stage | |
|  |  | Figure e1 | | (c) Consider use of a flow diagram | |
| Descriptive data | 14* | 9-10 Table1 | | (a) Give characteristics of study participants (eg demographic, clinical, social) and information on exposures and potential confounders | |
|  |  | 9, figure e1-e2 | | (b) Indicate number of participants with missing data for each variable of interest | |
|  |  | 9, figure e1-e2 | | (c) *Cohort study*—Summarise follow-up time (eg, average and total amount) | |
| Outcome data | 15* | *10*  figure e-2, figure 1 | | *Cohort study*—Report numbers of outcome events or summary measures over time | |
|  |  |  | | *Case-control study—*Report numbers in each exposure category, or summary measures of exposure | |
|  |  |  | | *Cross-sectional study—*Report numbers of outcome events or summary measures | |
| Main results | 16 | 11, 14-18  Table 2 &3 | | (*a*) Give unadjusted estimates and, if applicable, confounder-adjusted estimates and their precision (eg, 95% confidence interval). Make clear which confounders were adjusted for and why they were included | |
|  |  | 9-10 | | (*b*) Report category boundaries when continuous variables were categorized | |
|  |  |  | | (*c*) If relevant, consider translating estimates of relative risk into absolute risk for a meaningful time period | |
| Other analyses | 17 | 13 | | Report other analyses done—eg analyses of subgroups and interactions, and sensitivity analyses | |
|  | | | Discussion | | |
| Key results | 18 | 13 | | Summarise key results with reference to study objectives | |
| Limitations | 19 | 13-16 | | Discuss limitations of the study, taking into account sources of potential bias or imprecision. Discuss both direction and magnitude of any potential bias | |
| Interpretation | 20 | 13-16 | | Give a cautious overall interpretation of results considering objectives, limitations, multiplicity of analyses, results from similar studies, and other relevant evidence | |
| Generalisability | 21 | 16 | | Discuss the generalisability (external validity) of the study results | |
|  | | | Other information | | |
| Funding | 22 | 2,20-22 | | Give the source of funding and the role of the funders for the present study and, if applicable, for the original study on which the present article is based | |

*Give information separately for cases and controls in case-control studies and, if applicable, for exposed and unexposed groups in cohort and cross-sectional studies.

**Note:** An Explanation and Elaboration article discusses each checklist item and gives methodological background and published examples of transparent reporting. The STROBE checklist is best used in conjunction with this article (freely available on the Web sites of PLoS Medicine at http://www.plosmedicine.org/, Annals of Internal Medicine at http://www.annals.org/, and Epidemiology at http://www.epidem.com/). Information on the STROBE Initiative is available at www.strobe-statement.org.
